# Supplementary material for: ImmuneBuilder: Deep-Learning models for predicting the structures of immune proteins
Source: Commun Biol. 2023 May 29;6:575. doi: 10.1038/s42003-023-04927-7 (PMC10227038; doi:10.1038/s42003-023-04927-7)
Supplement: Supplementary file 2 — Supplementary Information [file 42003_2023_4927_MOESM2_ESM.pdf]

Supplementary Information

ImmuneBuilder: Deep-Learning models for predicting the structures of immune proteins.

Brennan Abanades[1], Wing Ki Wong[2], Fergus Boyles[1], Guy Georges[2], Alexander Bujotzek[2] and Charlotte M. Deane[1]

[1]Department of Statistics, University of Oxford, Oxford, UK.
[2]Large Molecule Research, Roche Pharma Research and Early Development, Roche Innovation Center Munich, Penzberg, Germany.

Supplementary Note 1 NanoBodyBuilder2

Nanobodies are single domain antibodies found in organisms such as camelids and sharks[1]. Their structure is similar to that of an antibody heavy chain variable region. However, the CDR-H3 loop in antibodies and nanobodies often take on very different conformations. While in antibodies the number of possible CDR-H3 conformations is limited by the light chain[2], the lack of light chain in nanobodies allows them to adopt a wider range of conformations[3]. To not bias the model towards light chain constrained CDR loop conformations, we trained separate models for antibody and nanobody structure prediction.

1.1 Methods

The model architecture, training procedure, model selection and structural refinement for NanoBodyBuilder2 is the same as for ABodyBuilder2 and is described in the main text. To train NanoBodyBuilder, 1935 nanobody structures were extracted from SAbDab[4] on the 10th of July 2022, fifty of which were randomly selected as a validation set. For the benchmark data, forty structures released between January and July 2022, with a resolution better than 2.3Å and resolved via X-ray diffraction were selected. It was ensured that there were no structures with the same sequence in the

train, tests and validation sets. A full list of these structures is given at https://github.com/oxpig/ImmuneBuilder.

1.2 Results

In this section we compare NanoBodyBuilder2, our nanobody-specific method, to three other tools used for nanobody structure prediction. We compared against two homology modelling methods (the original version of ABodyBuilder[5] and MOE[6]), and one general protein structure prediction method (AlphaFold2[7]). As a benchmark, we selected a non-redundant set of forty nanobody structures recently added to SAbDAb[4]. None of the benchmark structures are in the training or validation set for either of the deep learning methods used.

In Supplementary Table 1, we report results on the same metrics as were used for antibodies in the main text. The exceptions to this are metrics involving the light chain, such as heavy and light chain packing angles or light chain regional RMSDs.

Method	CDR1	CDR2	CDR3	Fw
ABodyBuilder	2.96	2.08	5.08	1.09
MOE	2.67	1.99	4.90	1.19
AlphaFold2	2.08	**1.35**	3.44	0.82
NanoBodyBuilder2	**1.98**	1.37	**2.89**	**0.79**

Method	$\chi 1$	$\chi 2$	$\chi 3$	$\chi 4$	E/B
ABodyBuilder	0.72	0.73	**0.54**	**0.63**	0.93
MOE	0.68	0.63	0.43	0.49	0.92
AlphaFold2	**0.78**	**0.74**	0.51	**0.63**	**0.94**
NanoBodyBuilder2	0.77	0.70	0.53	0.58	0.93

Method	Peptide bond	Clash	D-amino acid	Cis-bond
ABodyBuilder	49	0	8	21
MOE	0	7	6	0
AlphaFold2	0	0	0	0
NanoBodyBuilder2	0	0	0	0

Supplementary Table 1 Comprehensive benchmark between ABodyBuilder, MOE, AlphaFold2 and NanoBodyBuilder2 for predicting nanobody structures. In the first table, the mean RMSD to the crystal structure across the test set for each of the three CDRs and the framework is shown. In the second, we show the accuracy at modelling each of the first four torsion angles of the side chain (χ) and the accuracy at predicting whether a residue is exposed or buried (E/B). The third table shows the total number of the stereochemical errors found in the predicted structures. For a more a more in depth description of each metric, see the results section of the main text.

NanoBodyBuilder2 predicts the backbone structure of nanobodies with higher accuracy than all other benchmarked methods. The biggest improvement is in the CDR3 loop, which all methods model least accurately. NanoBodyBuilder2 also predicts the chi (χ) side chain angles with an accuracy comparable to AlphaFold2 and the original version of ABodyBuilder. In terms of speed, AlphaFold2 takes around one hundred times longer to generate a nanobody structure than any of the other methods.

In Supplementary Figure 1, the structure of an antibody heavy chain as predicted by ABodyBuilder2 is compared to the same chain as predicted by NanoBodyBuilder2. Although both models predict a similar structure for the framework, the predicted CDR-H3 conformation is very different.

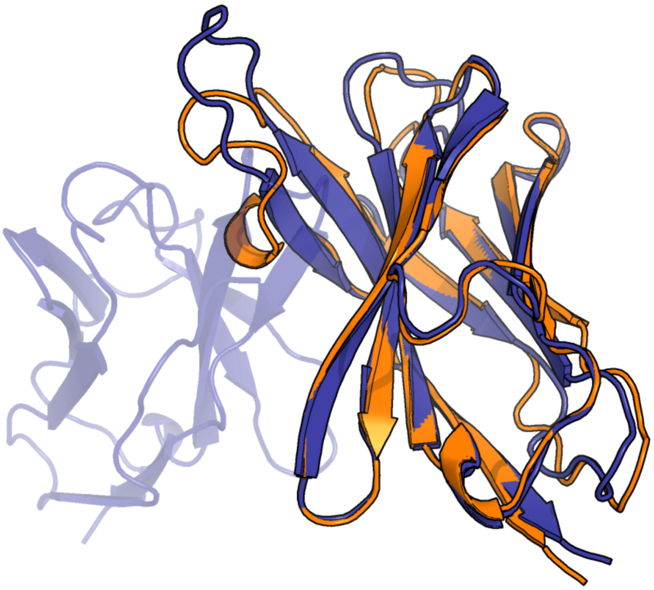

Supplementary Figure 1 Example of an antibody heavy chain as predicted by ABodyBuilder2 and NanoBodyBuilder2. The NanoBodyBuilder2 prediction is shown in orange and the ABodyBuilder2 prediction is shown in blue. While ABodyBuilder2 predicts the CDR-H3 to be in an erect conformation to avoid collisions with the light chain, NanoBodyBuilder predicts the loop to take a less compact form.

Supplementary Note 2 TCRBuilder2

The global structure of the variable domain in TCRs is similar to the antibody variable domain. However, antibody and TCR CDR loops tend to be of different lengths and mostly occupy distinct structural spaces[8]. As with nanobodies, we built a model for TCR structure prediction trained only on TCRs (TCRBuilder2).

2.1 Methods

The model architecture, training procedure, model selection and structural refinement for TCRBuilder2 is the same as for ABodyBuilder2 which is described in the main text. To train TCRBuilder2, the structures of 704 TCR variable domains were extracted from STCRDab[9] on 20th of May 2022, fifty

of which were randomly selected as a validation set. For the benchmark data, the structures of 21 alpha-beta TCRs with a resolution better than 3.5Å and released between January and August 2022 were selected. It was ensured that there were no structures with the same sequence in the train, test and validation sets.

2.2 Results

In this section we compare TCRBuilder2 against three other methods for TCR structure prediction. We benchmarked it against two homology modelling methods (RepertoireBuilder [10] and the original version of TCRBuilder [11]) and a general protein structure prediction method (AlphaFold-Multimer [12]). Additionally, we compared it to our antibody-specific method (ABodyBuilder2) to showcase the benefits of training only on TCRs.

Method	CDR-A1	CDR-A2	CDR-A3	Fw-A	CDR-B1	CDR-B2	CDR-B3	Fw-B
TCRBuilder	1.60	1.31	2.89	0.87	0.99	0.90	3.12	0.81
RepertoireBuilder	1.35	1.00	2.64	0.75	0.86	1.59	2.77	1.05
AlphaFold-M	**1.25**	0.96	**1.84**	**0.69**	0.75	0.65	1.94	0.82
ABodyBuilder2	3.49	6.57	3.14	2.89	3.27	3.77	3.48	3.65
TCRBuilder2	1.34	**0.93**	1.85	0.90	**0.74**	**0.63**	**1.93**	**0.67**

Method	HL	HC1	LC1	HC2	LC2	dc
TCRBuilder	4.80	3.07	2.17	4.03	1.77	0.32
RepertoireBuilder	8.34	3.09	2.60	3.66	2.79	0.45
AlphaFold-M	**2.95**	**1.45**	**1.97**	2.83	**1.51**	0.36
ABodyBuilder2	74.26	25.65	20.71	63.19	27.42	1.91
TCRBuilder2	3.32	1.88	2.80	**2.44**	1.94	**0.28**

Method	$\chi 1$	$\chi 2$	$\chi 3$	$\chi 4$	E/B
TCRBuilder	0.70	**0.69**	**0.53**	0.53	0.90
RepertoireBuilder	0.73	0.66	0.49	**0.54**	**0.92**
AlphaFold-M	**0.77**	**0.69**	0.50	0.52	**0.92**
ABodyBuilder2	0.58	0.52	0.36	0.39	0.83
TCRBuilder2	0.76	0.67	0.50	0.47	0.91

Method	Peptide bond	Clash	D-amino acid	Cis-bond
TCRBuilder	74	29	0	6
RepertoireBuilder	115	587	0	11
AlphaFold-M	0	0	0	0
ABodyBuilder2	0	0	0	0
TCRBuilder2	0	0	0	0

Supplementary Table 2 Comprehensive benchmark between ABodyBuilder2, TCRBuilder, AlphaFold-Multimer and TCRBuilder2 for predicting TCR structures. The top table shows the mean RMSD to the crystal structure across the test set for each of the six CDRs and frameworks. The CDRs and framework (Fw) regions are labelled A for the alpha chain and B for the beta chain. The second table shows the mean absolute error in each of the six ABangles [13]. In the third table, the accuracy at modelling each of the first four torsion angles of the side chain (χ) and the accuracy at predicting whether a residue is exposed or buried (E/B) is shown. The fourth table shows the total number of the stereochemical errors found in the predicted structures. For a more a more in depth description of each metric, see the results section of the main text.

ABodyBuilder2 fails to accurately model TCR structures, with the biggest errors being the modelling of the relative orientation between the alpha and beta chains. Both TCRBuilder2 and AlphaFold-Multimer predict structures with a comparable accuracy, outperforming RepertoireBuilder and the original version of TCRBuilder. In terms of run time, TCRBuilder2 takes about as long as ABodyBuilder2, RepertoireBuilder and TCRBuilder, which are all over a hundred times faster than AlphaFold-Multimer.

Supplementary Note 3 Heavy and light chain packing characterisation

To benchmark the accuracy of different methods at predicting the relative orientation between the VH and VL chains we use the absolute error in six values, taken from ABangle[13], that have been shown to fully characterise it. In Supplementary Figure 2, we provide a brief description on how each ABangle is defined. For a more in depth description, please see the original paper[13].

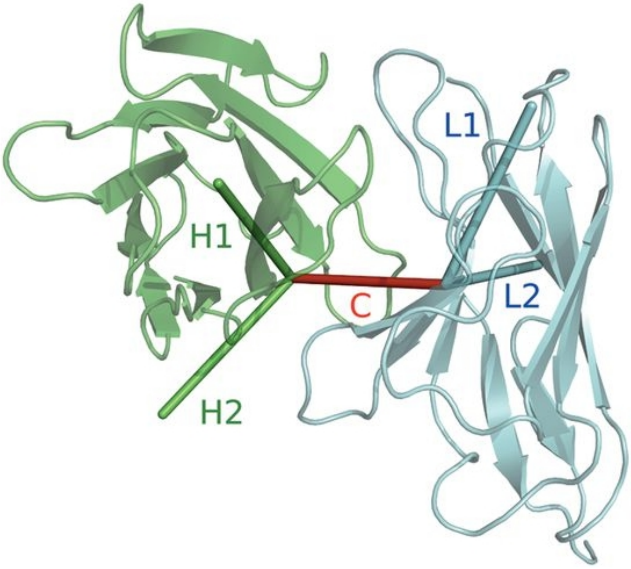

Supplementary Figure 2 Antibody structure with the five vectors used to calculate the 6 values used to define heavy and light chain packing. The distance dc is defined as the length of the vector C. The HL angle is defined as the torsion angle between the H1 and L1 vectors measured about C. The HC1, HC2, LC1 and LC2 angles are the angles between the H1, H2, L1 and L2 vectors and the vector C. Figure taken from[13].

Supplementary Note 4 Extended antibody prediction accuracy benchmark

4.1 Sequence identity to the train set does not determine prediction accuracy

Only antibody structures with an identical heavy and light chain sequence to those in the training set were excluded from the benchmark set. For each antibody in our benchmark, the most similar antibody in our training set has a sequence identity with values ranging from 62% to 99.5% (the latter having a single insertion in CDR-H3). Supplementary Figure 3 shows that having highly identical sequences in the training set does not necessarily improve the models ability to accurately predict CDR-H3.

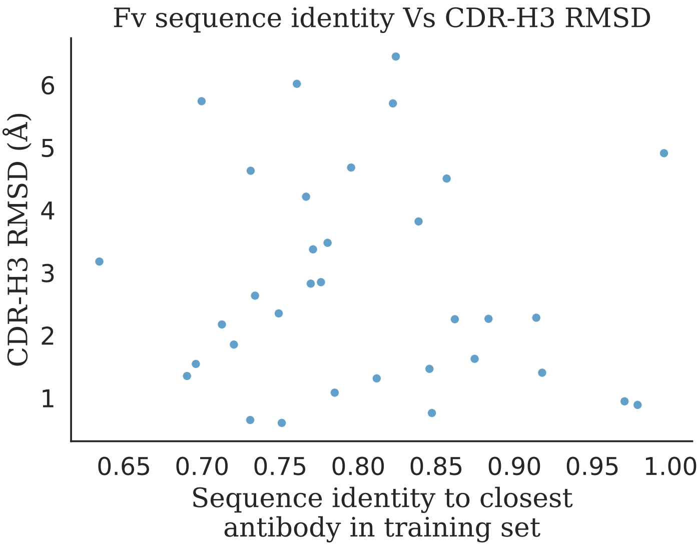

Supplementary Figure 3 CDR-H3 RMSD versus maximum sequence identity to the training set for ABodyBuilder2.

4.2 Performance of each method on individual antibodies in the benchmark

In Supplementary Figure 4, the CDR-H3 RMSD for each antibody in our test set is compared for each pair of benchmarked methods. In the majority of cases, ABodyBuilder2 is consistently better for most antibodies in the benchmark. The exception to this is AlphaFold-Multimer, where there appears to be a significant number of antibodies for which AlphaFold-Multimer significantly

outperforms ABodyBuilder and vice versa. It hence may be beneficial for some applications to combine the predictions from both methods. The individual RMSDs for each method for each CDR are given as tables in Supplementary Data 1.

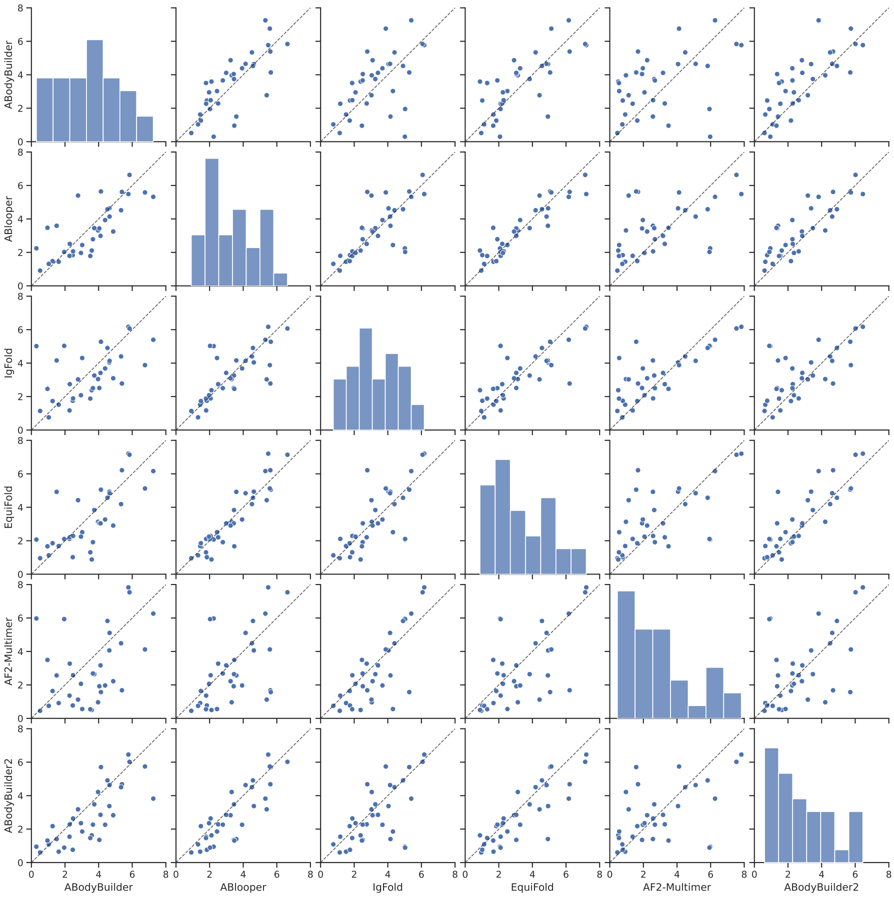

Supplementary Figure 4 Comparison of CDR-H3 RMSDs for each antibody in the test set for each of the benchmarked structure prediction methods. On the diagonal, a histogram showing the distribution of CDR-H3 RMSDs for each method is shown. Off diagonal, scatter plots comparing the ability of each method to model each CDR-H3 in the benchmark is shown. RMSDs are given in Angstroms (Å)

4.3 AlphaFold-Multimer template use

Throughout the paper we compare our methods against AlphaFold2 without the use of templates. In Supplementary Table 3 we compare the effect of using templates has on AlphaFold-Multimer predictions for the antibody benchmark. We only allow AlphaFold-Multimer to use templates from structures released

before the 1st of January 2022 to ensure it does not use any structures in our test set.

Method	CDR-H1	CDR-H2	CDR-H3	Fw-H	CDR-L1	CDR-L2	CDR-L3	Fw-L
AFM (no templates)	0.86	0.68	2.90	0.55	0.47	**0.40**	0.83	**0.54**
AFM (templates)	**0.84**	**0.67**	2.88	0.55	0.49	0.42	**0.81**	0.57
AB2	0.85	0.78	**2.81**	**0.54**	**0.46**	0.44	0.87	0.57

Supplementary Table 3 Comparison of performance when running AlphaFold-Multimer with or without templates for the antibody benchmark set.

As can be seen from Supplementary Table 3, the use of templates results in no significant improvement to the prediction accuracy of AlphaFold-Multimer on antibodies. However, we found that antibody structures generated using AlphaFold-Multimer with templates had a higher number of stereochemical errors. For the 34 antibodies in the benchmark set, AlphaFold-Multimer models were found to have two clashes, three unphysical peptide bonds and three D-amino acids. The non-template version of AlphaFold-Multimer generates none of these without any significant loss in accuracy, so it was used in all our benchmarks.

References

[1] Wesolowski, J. *et al.* Single domain antibodies: promising experimental and therapeutic tools in infection and immunity. *Medical microbiology and immunology* **198**, 157–174 (2009).

[2] Fernández-Quintero, M. L. *et al.* CDR loop interactions can determine heavy and light chain pairing preferences in bispecific antibodies. *Mabs* **14**, 2024118 (2022).

[3] Wang, N. *et al.* Conserved amino acid networks involved in antibody variable domain interactions. *Proteins: Structure, Function, and Bioinformatics* **76**, 99–114 (2009).

[4] Schneider, C., Raybould, M. I. & Deane, C. M. SAbDab in the age of biotherapeutics: updates including SAbDab-nano, the nanobody structure tracker. *Nucleic acids research* **50**, D1368–D1372 (2022).

[5] Leem, J., Dunbar, J., Georges, G., Shi, J. & Deane, C. M. ABodyBuilder: Automated antibody structure prediction with data–driven accuracy estimation. *MAbs* **8**, 1259–1268 (2016).

[6] Maier, J. K. & Labute, P. Assessment of fully automated antibody homology modeling protocols in molecular operating environment. *Proteins: Structure, Function, and Bioinformatics* **82**, 1599–1610 (2014).

[7] Jumper, J. *et al.* Highly accurate protein structure prediction with AlphaFold. *Nature* **596**, 583–589 (2021).

[8] Wong, W. K., Leem, J. & Deane, C. M. Comparative analysis of the CDR loops of antigen receptors. *Frontiers in immunology* **10**, 2454 (2019).

[9] Leem, J., de Oliveira, S. H. P., Krawczyk, K. & Deane, C. M. STCRDab: the structural T-cell receptor database. *Nucleic acids research* **46**, D406–D412 (2018).

[10] Schritt, D. *et al.* Repertoire builder: high-throughput structural modeling of b and t cell receptors. *Molecular Systems Design & Engineering* **4**, 761–768 (2019).

[11] Wong, W. K. *et al.* TCRBuilder: multi-state T-cell receptor structure prediction. *Bioinformatics* **36**, 3580–3581 (2020).

[12] Evans, R. *et al.* Protein complex prediction with AlphaFold-Multimer. *BioRxiv* (2021).

[13] Dunbar, J., Fuchs, A., Shi, J. & Deane, C. M. ABangle: characterising the VH-VL orientation in antibodies. *Protein Engineering, Design & Selection* **26**, 611–620 (2013).
