# Supplementary material for: ImmuneBuilder: Deep-Learning models for predicting the structures of immune proteins
Source: Commun Biol. 2023 May 29;6:575. doi: 10.1038/s42003-023-04927-7 (PMC10227038; doi:10.1038/s42003-023-04927-7)
Supplement: Supplementary file 3 — Description of Additional Supplementary Files [file 42003_2023_4927_MOESM3_ESM.pdf]

Description of Additional Supplementary Files

File name: Supplementary Data 1
Description: RMSD values for each CDR loop for each method benchmarked on antibody structure prediction.

File name: Supplementary Data 2
Description: RMSD values for each CDR loop for each method benchmarked on nanobody structure prediction.

File name: Supplementary Data 3
Description: RMSD values for each CDR loop for each method benchmarked on TCR structure prediction.

File name: Supplementary Data 4
Description: Data to replicate Figure 3 from the manuscript.
